# Research on Public Opinion Propagation of Emergency Reversal Based on Machine Learning

**DOI:** 10.1007/s44196-023-00254-1

**Published:** 2023-05-08

**Authors:** Xianwen Wu, Zixuan Liu

**Affiliations:** College of Public Management and Law, Hunan Agricultural University, Changsha, Hunan China

**Keywords:** Unexpected events, Public opinion reversal, Influencing factors: driving paths, QCA model

## Abstract

As an empirical case, this study takes 30 sudden reversal events as examples, combined with the theory of actor network, and explores the four influencing factors of public opinion subjects-netizens and opinion leaders, public opinion objects-events, public opinion carriers-media, and public opinion guides-government in public opinion. The complex combinatorial effects arise during the reversal process. This study verifies the combination of three parallel and equivalent driving paths that lead to the multi-center reversal of public opinion, the opinion leader–media dual-driven path, the opinion leader–media–government multi-driven path, and the media–government dual-driven path. It is concluded that the public should improve their media literacy and maintain a rational return; the media, as “gatekeepers”, need to improve their own awareness and build an objective issue framework; the government needs to establish active communication awareness, and supervision and guidance should go hand in hand.

## Introduction

In recent years, the phenomenon of reversal of public opinion on the Internet of unexpected events has become more and more frequent, affecting the social order in cyberspace and bringing great negative impact to the national public opinion security and social harmony. “Public opinion reversal” is a new form of public opinion derived from online public opinion in recent years, and the destructive effect of this new public opinion is even stronger. Every reversal of public opinion may lead to rumors and online violence, and if there is a lack of proper management and guidance, it may easily cause public crisis of public opinion or even social disorder. Therefore, the phenomenon of reversal of public opinion caused by unexpected events urgently needs further exploration and research.

## Literature Review and Analysis Framework

### Literature Review

As a special form of online opinion reversal, theories and methods related to online opinion evolution are worth studying and learning. First, from the perspective of online opinion formation, Lucinda et al. explored the influence of events on public opinion information from the event itself [1]; Schulz and Roessler explained the formation process of online public opinion from the perspective of the perception of public opinion atmosphere [2]; Kwon et al. studied rumors in online public opinion and concluded that online rumors can influence public opinion and sentiment and, thus, influence the formation of online public opinion [3]. Arias analyzed the impact of the use of self-media on political elections and proposed that online public opinion is essentially a manifestation of the aggregation of netizens' emotions, which will make the public think about the definition and perception of liberal democracy [4]. Some scholars have also studied from the perspective of online public opinion governance, and Gabrielle et al. proposed that an online public opinion ecology should be constructed to promote a consensus of responsible participation among subjects [5].Suskevics et al. used a semi-structured interview method to interview stakeholders and concluded that collaborative participation of relevant participants in governance can provide effective assistance in public opinion management [6]. Provan and Kenis argued that effective online opinion governance requires timely response to various needs of the concerned subjects [7].

For the phenomenon of public opinion reversal, to study its influencing factors, it is also very helpful to draw on the influencing factors of online public opinion evolution. The formation and propagation of online public opinion is a complex process that is influenced by a variety of factors. Wang et al. studied the generation of enterprise online public opinion heat from four dimensions: enterprises, Internet users, media, and government, and finally concluded that three modes such as internal and external linkage, internal domination, and external constraint would trigger high heat online public opinion [8]. Scholar Gao used fuzzy set qualitative comparative analysis to study online public opinion backlash, and the study showed that event type, audience and information feedback are the key influencing factors of its public opinion backlash [9]. Cheng et al. studied the relationship between topic relevance and public opinion elicitation using public health emergencies, and the results pointed out that the degree of topic relevance among public health emergencies was significantly and positively correlated with the intensity of resonance of online public opinion [10]. Scholars Li et al. explored the trends and characteristics of the evolution of online public opinion on animal epidemics, and pointed out that government intervention is crucial to public opinion [11]. Lan studied the influence of online rumors on online public opinion and introduced the logistic population growth model and differential equation theory to propose that online rumors fuel online public opinion to reach its peak [12]. Scholar Qin used the DEMATEL method to scientifically identify and evaluate the main factors influencing the hotness of online public opinion from the perspective of information ecology, and concluded that the government's crisis handling ability and guidance, opinion leaders, topic types, the participation of water army and participation response index are the key factors influencing the evaluation of online public opinion hotness [13].

Facing the online public opinion of emergencies, there are certain differences in the handling and response among countries. Some of them focus on the timeliness of government response for the governance of public opinion in emergencies, for example, Brauchler B, from the government's perspective, believes that the biggest cause of the current online public opinion crisis is due to the government’s ineffective response, and therefore proposes that when facing a public opinion crisis, the government should take measures as soon as possible to shift from being reactive to being proactive [14]. Scholars such as KwonK H focus on the influence of online 14public opinion on policies, laws, and regulations on online public opinion, and provide a comprehensive analysis of its development trend and causes, and argue that the cause of its generation lies in the government, and therefore, the regulation of online public opinion should be strengthened from the perspective of online governance [15]. Bastos Filip et al. argued that unexpected events can undermine the public opinion security system and advocated for providing more policy support and legal guidance to provide appropriate rights and interests for injured parties [16].

In general, the existing studies on reversal of online public opinion provide a good reference for this paper, but there are still two shortcomings: first, few studies on reversal of public opinion on unexpected events mention the inner mechanism, and the driving path of reversal of online public opinion on unexpected events needs to be clarified; second, few existing studies explore the interaction between influencing factors that lead to polycentric reversal of public opinion; finally, most studies on reversal of public opinion on unexpected events use single-case analysis and lack cross-case and holistic perspectives. Finally, most studies on public opinion reversal use single-case analysis and lack cross-case and holistic perspectives. Therefore, this paper uses actor networks and holism as perspectives to qualitatively compare 30 reversals and identify the combined effects of influencing factors, so as to derive the driving path of reversal of public opinion on the Internet of sudden events, and then provide reference for the government to manage reversed public opinion.

### Analysis Framework

**Fig. 1:**
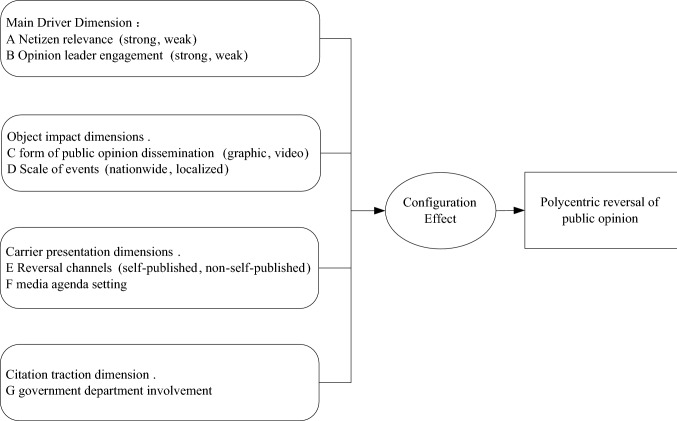
Analytical model: the grouping effect of actor networks on opinion reversal

First, we summarize the existing literature and conclude that the participating subjects of online public opinion are mainly divided into netizens, the media, the government, and events, and the existing studies show that all four of them interact and intertwine with each other to influence the development and evolution of online public opinion. Actor network theory is a social science idea proposed by French sociologists Callon and Latour in the mid-1980s. The idea is that society is a dynamic network of human and non-human actors who are connected to each other. The non-human actors mainly include institutions, cultural concepts, technology and other existences that can change the state of things, and in this network, the actors with life characteristics and those without life characteristics are equal in status, interacting and connecting with each other. Actor network theory advocates a role relationship between non-human and human actors, which makes people pay attention to non-human actor factors when studying issues. In the study of online public opinion issues, the actor network should include not only netizens, media, and governments but also factors related to the events themselves. Combining with the actor network theory to analyze each participating subject in public opinion reversal, the actor network of public opinion reversal is finally divided into public opinion subject, public opinion object, public opinion carrier, and public opinion elicitor. In the actor network, Internet users who are the subjects of public opinion have a driving role in the actor network and can dominate the direction of public opinion and influence the dynamics of events [17]. The public opinion carrier mainly reflects the presentation of the event, i.e., showing the context of the event. The media plays the role of a good information channel and is the key to the reversal of public opinion to the truth. The government plays an important role in this actor network as the lead of public opinion, and its intervention and guidance can speed up the presentation of the truth of the incident. Generally speaking, the earlier and more timely the government intervention, the less the reversal of public opinion. For the event itself, it mainly plays the role of an object in the network of actors, which is mainly reflected in the influence of the event itself. It mainly includes the various forms of communication and the scale of the event. The relationship among these actors will change the trend of public opinion reversal. Therefore, based on the group perspective and considering factors such as netizens, media, netizens, and events at the same time, it helps to explore the relationship between driving path and public opinion reversal in detail, and the specific research framework is drawn as shown in Fig. 1.

## Research Methods

### QCA Method

Using Qualitative Comparative Analysis (QCA), which was founded by Charles C. Ragin in 1987, the holistic perspective analysis method that discovers the relationship between elemental groupings and outcomes through set theory and Boolean algebra, takes the public opinion reversal event as the research object, the influence of the public opinion reversal factors as the condition variables, and whether the public opinion is polycentric reversal as the outcome variable[18]. This paper adopts the fsQCA method to explore the core influencing factors of public opinion reversal outcomes and their driving paths. fsQCA adopts a holistic perspective, conducts cross-case comparative analysis, and is dedicated to exploring the causal complexity of which groups of conditional elements cause the emergence of expected outcomes and which groups cause the absence or non-existence of expected outcomes. The combination of elements in online opinion reversal forms different paths, so it is particularly suitable for research using the fsQCA method. The fsQCA approach assumes that the greater the number of condition variables set for a research phenomenon, the greater the risk of "individualized" interpretation of the cases, in other words, it is better to keep the number of conditions relatively small in the study design to achieve a good balance between the number of conditions and the number of cases. According to the practice of fsQCA, usually 4–7 condition variables are selected, and the corresponding number of cases is a small- and medium-sized sample (10–40 cases). Therefore, this paper refines the seven conditional variables based on the four dimensions derived from the analysis of actor networks in sudden-onset online opinion reversal to fit with the selected number of 30 cases. The QCA method is used to quantitatively evaluate the condition variables, explore the necessary conditions that lead to the polycentric reversal of public opinion and the relationship between the conditions, and use them as a basis to identify the driving paths of the reversal of public opinion on the Internet of unexpected events.

### Data Sources

This paper selects the reversal events in the past five years as the cases of online opinion reversal research. By summarizing the major news reversal events on the Zhiwei data platform, we selected typical public opinion reversal events to enter the research case database, including “Shuanghuanglian inhibited 2019-nCoV events”, “Henan Zhoukou baby loss case,” etc. For the basic overview of the cases, please refer to In general, the cases can meet the basic requirements for comparative analysis of QCA: first, they all experienced the process of reversal of public opinion; they are all rooted in the same context; and they have strong comparability in terms of important influencing factors and driving paths of reversal of public opinion, meeting the similarity required by QCA cases. Second, there are strong differences among the cases, and the cases involve a variety of social hot issues, covering various fields such as doctor-patient relationships and government-public relations, which satisfy the heterogeneity of QCA; finally, this paper covers both “positive” cases with a single center reversal of public opinion and “negative” cases with a single center reversal of public opinion, which is consistent with the two-sidedness of the case results (Table 1).

**Table 1:** Case database of online opinion reversal events

No.	Event name	No.	Event name
1	Yuan Longping's misrepresented death	16	Simba’s live broadcast with goods reverses public opinion
2	Female passenger dies in crash	17	“Nurse aided to Hubei did not receive preferential treatment” incident
3	Indecent video of students of Heilongjiang University of Science and Technology	18	Taobao store “Guo Xiaoyun" event
4	Qinhuangdao boy rescue sacrifice incident	19	Food Safety Issues in the Experimental School of Chengdu No. 7 Middle School
5	Poverty alleviation cadres angry at the lazy father and son incident	20	Saya assaults pregnant woman
6	Zhang Wenhong was attacked by Internet violence	21	Wang Fengya incident
7	Alibaba female employees were assaulted	22	Female doctor's suicide in Deyang
8	Hongxing Erke Donated “Disaster Area in Henan”	23	Chongqing bus crash incident
9	Shuanghuanglian inhibited 2019-nCoV events	24	Yueqing children “lost contact”
10	Bao Yuming incident	25	Henan College Entrance Examination Package Transfer Case
11	“Guangzhou Teacher Suspected of Corporally Punishing Students” Incident	26	Hangzhou nanny arson case
12	“Yuanyang public opinion” outbreak events	27	“Fighting Orphans” Incident
13	Hangzhou woman missing case	28	Yulin pregnant woman falling incident
14	Tencent sues Laoganma incident	29	South causeway bookstore Park Road thatched hall staged drama
15	Luo champion incident	30	Luo Yixiao incident

### Description of Variables’ Selection

#### Outcome Variables

The measurement of the evolution of online public opinion on emergencies is a very complicated issue in itself, and this is especially true for public opinion reversal as a type of branch of online public opinion. Reversal of public opinion means two or more constructions of public opinion, i.e., the original convergent opinion is rapidly transformed into the opposite opinion and forms an absolute advantage. The core research question of this study is to reveal the core influencing factors of public opinion reversal outcomes and their driving.

#### Antecedent Conditions

The “actor theoretical framework of online opinion reversal” built in the previous section constitutes the basic scope for selecting the conditional variables in this paper, and seven factors in the four dimensions of opinion subjects, opinion objects, opinion carriers and opinion elicitors are finally selected as the conditional variables.

1. Internet users' relevance. As we all know, Internet public opinion is the opinions, attitudes and emotions of netizens on events, and the main body is netizens. When the events are closely related to the personal interests of netizens, they will be very active and participate in the public opinion actively, and the public opinion will rise. Therefore, this paper defines netizen relevance as low relevance when the events target ordinary social phenomena and problems; when the events involve life and property safety, netizen concern will rise and be of high relevance. In general, the higher the relevance to netizens, the more likely the events are to be reversed.
2. Involvement of opinion leaders. Opinion leaders are opinion subjects with many fans, whose words and deeds can drive the emotions of Internet users and have strong influence and appealing power. Relevant studies have shown that the participation of opinion leaders can influence the trend of the whole public opinion and, thus, drive the development of public opinion. Especially in the frequent reversal of public opinion, the high participation of opinion leaders will directly promote the peak of public opinion. Therefore, this paper selects the number of fans greater than 100,000 as the standard, and quantifies the two levels of high and low, with the number of fans of opinion leaders greater than or equal to 100,000 being strong participation and the number of fans less than 100,000 being low participation.
3. Forms of public opinion dissemination. The forms of public opinion dissemination are mainly divided into two types: graphic and video. In the case of reversal of public opinion, netizens and major self-publishers will choose to release information on the event to attract people's attention, which involves the problem of information falsification.
4. The scale of the event. The scale of the event reflects the extent of the outbreak and impact of the event, which can be divided into a national scope and a local scope. Different events have different social audiences, and the interests of people are naturally different.
5. Reversal channels. Online public opinion reversal is a product of the new media era, and the development of new media technology provides very good conditions for its emergence. The media is mainly divided into self-media and non-self-media; self-media is mainly for individuals; non-self-media is divided into governmental media and social media; the function of governmental media is to correctly guide public opinion and relieve the negative emotions of the online public, which is an important channel for public opinion reversal.
6. Media agenda setting. In the case of the reversal, many media outlets will selectively report on the incident before the government department announces the investigation results to attract more audience attention and traffic, thus achieving the purpose of obtaining clicks. Therefore, this paper includes the variable of agenda setting by the media in the impact study.
7. Government departments intervene in the investigation. The involvement of government departments in the investigation is the core strength of the government's ability to manage the reversal of public opinion, and they have the obligation to release information and disclose the truth to the public in a proactive and timely manner. At the same time, government departments, as controllers of online public opinion information, also have the responsibility to transform the situation and reduce the harm. The information in the reversal of public opinion is complicated, and most of the information is even false news at the beginning, so it is difficult for the public to distinguish the true from the false. It is necessary for the government departments to come forward to investigate the truth and return the truth to the incident and the public, so as to correctly guide the public opinion and make the online public opinion reversal to the truth.

### Variable Assignment and Truth Table Construction

#### Variable Assignment

This paper uses the clear set of qualitative comparative analysis in the qualitative comparative analysis, so the “dichotomous attribution principle” must be observed when assigning values to the variables. Therefore, this paper assigns the outcome variable “number of reversals” and the conditional variables “relevance of netizens”, “involvement of leaders”, “form of public opinion”, “scale of events”, and “size of events”. The outcome variable “number of reversals” and the conditional variables “relevance of netizens”, “involvement of leaders”, “form of opinion dissemination", “scale of events”, “reversal channels”, “media agenda setting”, and “involvement of government departments” are dichotomously assigned. The dichotomous assignment of “Involvement”, i.e., a value of 1 means occurrence or existence, and a value of 0 means no occurrence, no occurrence or no existence, is indicated by “ ~ ”, as shown in Table 2.

**Table 2:** Variable assignment rule table

Theoretical dimensions	Condition variables	Variable descriptions	Coding rules
Subjects of public opinion	Netizen relevance (NET)	Relevance to the immediate interests of Internet users	Strong, 1; weak, 0
	Leader opinion engagement (OPL)	The number of fans is greater than 100,000 as the standard	Greater than or equal to 100,000, 1; less than 100,000, 0
Public opinion object	Forms of opinion dissemination (FOD)	Graphic form, video form	Graphic form, 1; video form, 0
	Event scale (ES)	Nationwide, local scope	Nationwide, 1; Domestic local, 0
Public opinion carrier	Reversal channels (RS)	Self-publishing, non-self-publishing	Non-self-publishing, 1; Self-publishing, 0
	Media Agenda Setting (MAS)	Agenda setting or not	Yes, 1; No, 0
Opinion citation body	Government department intervention (GOV)	Is the government department involved	Yes, 1; No, 0

#### Truth Table Construction

After defining the conditional and outcome variables, the selected 30 cases are summarized, organized and coded to obtain a dichotomous table of the case base of opinion reversal events, as shown in Table 3.

**Table 3:** Original database of public opinion reversal events

Case	NET	OPL	FOD	ES	RS	MAS	GOV	RESULT
Case1	1	1	1	1	1	0	0	0
Case2	1	1	1	0	0	1	1	1
Case3	0	0	0	0	1	0	0	0
Case4	1	1	1	0	1	0	0	1
Case5	1	1	0	0	1	1	0	0
Case6	0	0	1	0	1	0	0	0
Case7	1	0	1	0	1	1	1	1
Case8	1	1	1	1	0	0	0	0
Case9	1	1	1	1	1	0	0	0
Case10	1	1	1	0	1	1	1	1
Case11	1	1	0	0	1	1	1	0
Case12	1	1	1	0	1	0	1	1
Case13	1	1	1	0	1	1	1	1
Case14	0	0	1	0	1	0	1	0
Case15	0	0	1	0	0	0	0	0
Case16	1	1	0	0	1	0	1	0
Case17	0	0	1	0	0	0	0	0
Case18	0	0	1	0	1	0	0	0
Case19	1	1	1	0	1	0	1	1
Case20	1	1	1	0	0	1	1	1
Case21	1	1	1	0	1	1	1	1
Case22	1	1	1	0	1	0	1	1
Case23	1	1	1	0	1	1	1	1
Case24	1	1	1	0	1	0	1	1
Case25	1	1	1	0	1	1	1	1
Case26	1	1	1	0	1	1	1	1
Case27	0	0	0	1	1	0	1	1
Case28	1	1	1	0	1	0	1	1
Case29	0	0	1	0	0	0	0	0
Case30	1	1	1	0	1	0	0	1

The qualitative analysis software Tosmana 1.6 was used to integrate the original data table into a clear set truth table with the outcome variable of opinion polycentric inversion. The truth table of Boolean algorithm contains conditional variables, different logical combinations of all conditional variables, outcome variables, and case-related information. The truth table can observe both the multiple conditions that lead to the occurrence or non-occurrence of the outcome variable and the relationship between the effects of the condition variables, as well as the frequency of occurrence of the effects between different conditions and the number of cases included, so as to derive how these combinations of condition variables lead to the occurrence or non-occurrence of the study outcome. First, in the process of refining the truth table, the threshold for selecting the number of cases was set to 1, at which point condition combinations for which no cases could be observed would be eliminated from the truth table. Then, the coincidence threshold was set to 0.8, and the results obtained are shown in Table 4.

**Table 4:** Truth table

NET	OPL	FOD	ES	RS	MAS	GOV	Result	Number
0	0	0	0	1	0	0	0	1
0	0	0	1	1	0	1	1	1
0	0	1	0	0	0	0	0	3
0	0	1	0	1	0	0	0	2
0	0	1	0	1	0	1	0	1
1	0	1	0	1	1	1	1	1
1	1	0	0	1	0	1	0	1
1	1	0	0	1	1	0	0	1
1	1	0	0	1	1	1	0	1
1	1	1	0	0	1	1	1	2
1	1	1	0	1	0	0	1	2
1	1	1	0	1	0	1	1	5
1	1	1	0	1	1	1	1	6
1	1	1	1	0	0	0	0	1
1	1	1	1	1	0	0	0	2

Source: generated by fsQCA software

## Analysis Results

### Univariate Necessity Analysis: Key Influencing Factors of Public Opinion Reversal

The analytical logic of the QCA method suggests that there are multiple concurrent, nonlinear relationships among the condition variables, i.e., the generation of the same outcome can be composed of multiple factors. If A and B occur simultaneously, they generate Y (that is, A*B + Y), C and D can also generate Y (that is, C*D + Y), then A*B + C*D → Y. Also, a condition always occurs when some result is generated, then this condition is the necessary condition for that result to be generated for example, A*B + A*C = Y. In the case of Y, there is necessarily a condition A, therefore, A is the the premise of the result.

Before conducting a clear-set qualitative analysis, it is necessary to test which of the selected influences act as critical factors leading to the occurrence of the outcome. Therefore, in this study, the seven identified conditional variables and their inverse values were subjected to univariate necessity tests for both positive and negative outcome variables. The resulting outcome indicators are generally Consistency and Coverage, where Consistency is used to explain the probability that the condition variable leads to the outcome variable, when Consistency ≥ 0.8, when the condition variable appears as a sufficient condition for the outcome variable, and when Consistency ≥ 0.9, when the condition variable becomes necessary to lead to the outcome. The coverage indicates the strength of the explanation of the outcome variable by the condition variable, that is, how many cases are available in the case database to explain the relationship between the two variables [19]. Also “~” indicates “not”, i.e., the opposite value. The calculation formula is shown below:

$$ {\text{Consistency}}\left( {Xi \le Yi} \right) = \frac{{\sum {[\min (Xi,Yi)]} }}{{\sum {Xi} }}, $$

$$ {\text{Coverage}}\left( {Xi \le Yi} \right) = \frac{{\sum {[\min (Xi,Yi)]} }}{Yi}. $$

The results of univariate necessity analysis of a total of seven variables and their respective opposite values of netizens' relevance, leaders' opinion participation, opinion dissemination form, event scale, reversal channel, media agenda setting, and government department involvement in investigation are shown in Table 5. The results of the analysis of the variables leading to the polycentric reversal of public opinion show that “netizens’ relevance”, The consistency of “form of public opinion dissemination” and “ ~ event scale” is greater than 0.900, indicating that these three single variables are necessary as the conditions for the polycentric reversal of public opinion and have strong explanatory power. Their corresponding coverage ratios are 0.72727, 0.64000, and 0.61539, respectively, indicating that all three have strong explanatory power as single variables for the polycentric inversion of public opinion.

**Table 5:** Univariate tests of necessity

Conditional variables	Resulting variable = multi-center inversion		Resulting variable = single center inversion	
	Consistency	Coverage	Consistency	Coverage
Netizen relevance	0.94118	0.72727	0.46154	0.27273
~ Netizen relevance	0.05882	0.12500	0.53846	0.87500
Opinion leader engagement	0.88235	0.71429	0.46154	0.28571
~ Opinion leader engagement	0.11765	0.22222	0.53846	0.77778
Forms of public opinion dissemination	0.94118	0.64000	0.69231	0.36000
~ Forms of public opinion dissemination	0.05882	0.20000	0.30769	0.80000
Event scale	0.05882	0.25000	0.23077	0.75000
~ Event scale	0.94118	0.61539	0.76923	0.38462
Reversal channels	0.88235	0.62500	0.69231	0.37500
~ Reversal channels	0.11765	0.33333	0.30769	0.66667
Media agenda setting	0.52941	0.81818	0.15385	0.18182
~ Media agenda setting	0.47059	0.42105	0.84615	0.57895
Government department intervention	0.88235	0.83333	0.23077	0.16667
~ Government department intervention	0.11765	0.16667	0.76923	0.83333

Data source: Translated and compiled by the author from the output of data analysis software

It is worth noting that the consistency of opinion leaders' participation, reversal channels and government departments’ involvement in the investigation is 0.8, which is close to the necessary condition test of 0.9, constituting a sufficient condition for polycentric reversal of public opinion, indicating that the three factors also play a significant role in the process of polycentric reversal of public opinion. On the other hand, the results of the univariate necessity analysis for the outcome variable of monocentric reversal show that the consistency of only “~ media agenda setting” is 0.84615, which is greater than the sufficient condition test, indicating that when the public opinion of an emergency event is monocentric reversed, it is nearly 85% likely that the media did not set the agenda for the event when it was reported in the news. agenda setting.

#### Strong Relevance of Netizens is a Necessary Condition for Polycentric Reversal of Public Opinion

According to the results of the univariate necessity analysis, among the variables of the main dimension of public opinion, the consistency of positive “netizen relevance” reached 0.94118, indicating that the strong relevance of netizens is a necessary condition for the reversal of public opinion; the coverage rate is 0.72727, indicating that in the case Nearly 73% of the cases in the database contain the condition of strong relevancy of netizens; The consistency of “involvement of opinion leaders” is 0.88235, which is a sufficient condition for the interpretation of the results but not a necessary condition, so it is not a core influencing factor. This paper will interpret the role of opinion subjects in the process of reversal of public opinion based on the perspective of “netizen relevance”.

The reversal of public opinion occurs because external information stimulates netizens' internal emotions, and is also a derivative of the evolution of online public opinion, or an “anti-silence spiral” phenomenon. In the process of reversal of public opinion, unexpected events with a high degree of relevance to netizens can attract more attention from the society, and events with topicality are generally prone to reversal. This is equivalent to the social combustion substance generated by public opinion, the intrinsic emotion of netizens is equivalent to the accelerant, and the unexpected event is the ignition temperature. In the past, almost all of the hot topics that triggered public opinion across the Internet were public topics of social concern.

#### Graphic Communication Form is a Necessary Condition for Polycentric Reversal of Public Opinion

According to the results of univariate necessity analysis, among the variables of the opinion object dimension, the consistency of positive "form of opinion dissemination" reaches 0.94118, which meets the necessity criterion, so that graphic form dissemination is the core influencing factor of opinion reversal; its coverage rate reaches nearly 64%, which means that 65% of the original data case database can explain the result that graphic form dissemination leads to opinion reversal. The coverage rate reaches nearly 64%, indicating that 65% of the cases in the original data base can explain the result that graphic form dissemination leads to the polycentric reversal of public opinion. This section then interprets the role of graphic communication in the reversal of public opinion.

The presentation forms of information in the process of public opinion reversal are diversified and mainly divided into two forms: picture and text, and video. Among them, the form of pictures combined with text is more easily accepted by readers and more intuitive. On the one hand, in the current information flood era, information is mostly in fragmented form, and people have gradually developed the habit of using fragmentation to read, so for information, the concise and intuitive way of presenting information in graphic form is more in line with this habit; while for video form, it is often necessary to click and download to present. On the other hand, the reversal of public opinion is mostly caused by false news reports, in which the text is highly readable and inflammatory, coupled with very realistic pictures to support it, creating an illusion of “truth with pictures”, and it is easy for the first issuer to blur the information when describing the event or phenomenon in order to attract attention, and it is also easy to avoid and deliberately fabricate false rumors in the text expression, thus laying the foundation for the reversal of public opinion. At the same time, as far as the cost and technical requirements of falsification are concerned, the graphic form is relatively low and almost everyone can do it, thus greatly reducing the accuracy and authenticity of the information.

#### Local Scope Outbreak is a Necessary Condition for Polycentric Reversal of Public Opinion

According to the results of univariate necessity analysis, among the variables of the opinion object dimension, the consistency of positive "form of opinion dissemination" reaches 0.94118, which meets the necessity criterion, so that graphic form dissemination is the core influencing factor of opinion reversal; its coverage rate reaches nearly 64%, which means that 65% of the original data case database can explain the result of opinion reversal due to graphic form dissemination. The coverage rate reaches nearly 64%, indicating that 65% of the cases in the original data base can explain the result that graphic form dissemination leads to the polycentric reversal of public opinion. The next part of this paper explains the role of graphic communication in the reversal of public opinion.

On the one hand, the statistics of public opinion reversal events in recent years show that most of the reversal events are local emergencies, and the events can be first disseminated in local media more quickly after they occur. Due to their own advantages, local media, as sources of information, are prone to report misinformation about the events in order to attract public attention and clicks. On the other hand, local breaking news information has a smaller audience and spreads faster, so when the event is reversed it can be spread quickly, thus increasing the number of reversals. In addition, it has also been proved that in terms of the intensity of response to breaking news, central media tend to be more responsive than local media, and report and respond to news facts more authoritatively.

### Path Analysis of Condition Combination of Public Opinion Reversal

QCA analysis provides three solutions for the study, which are Complex Solution, Intermediate Solution and Parsimonious Solution, as shown in Tables 6 and 7. QCA outputs the resultant outcome variables as the combined paths of public opinion polycentric and monocentric inversions, respectively, among which a total of four complex paths, four intermediate paths and eight parsimonious paths are finally output for public opinion polycentric inversion results.

**Table 6:** Multi-center reversal path of public opinion

Path type	Condition	Consistency	Coverage
Complex path	NET*OPL*FOD* ~ ES*RS* ~ MAS	1	0.411765
	NET*OPL*FOD* ~ ES*MAS*GOV	1	0.117647
	NET*FOD* ~ ES*RS*MAS*GOV	1	0.0588235
	~ NET* ~ OPL* ~ FOD*ES*RS* ~ MAS*GOV	1	0.0588235
Intermediate paths	NET*OPL*FOD* ~ ES*RS* ~ MAS	1	0.411765
	NET*OPL*FOD* ~ ES*MAS*GOV	1	0.470588
	NET*FOD* ~ ES*RS*MAS*GOV	1	0.411765
	~ NET* ~ OPL* ~ FOD*ES*RS* ~ MAS*GOV	1	0.0588235
Minimalist path	NET*FOD* ~ ES	1	0.941176
	~ FOD*ES	1	0.0588235
	~ OPL*ES	1	0.0588235
	~ NET*ES	1	0.0588235
	ES*GOV	1	0.0588235
	~ OPL* ~ FOD*GOV	1	0.0588235
	~ NET* ~ FOD*GOV	1	0.0588235

Data source: Translated and compiled by the author from the output of data analysis software

**Table 7:** Public opinion monocentric reversal path

Path type	Condition	Consistency	Coverage
Complex Path	~ NET* ~ OPL*FOD* ~ ES* ~ MAS* ~ GOV	1	0.384615
	~ NET* ~ OPL* ~ ES*RS* ~ MAS* ~ GOV	1	0.230769
	~ NET* ~ OPL*FOD* ~ ES*RS* ~ MAS	1	0.230769
	NET*OPL*FOD*ES* ~ MAS* ~ GOV	1	0.230769
	NET*OPL* ~ FOD* ~ ES*RS*MAS	1	0.153846
	NET*OPL* ~ FOD* ~ ES*RS*GOV	1	0.153846
Intermediate paths	~ NET* ~ OPL*FOD* ~ ES* ~ MAS* ~ GOV	1	0.384615
	~ NET* ~ OPL* ~ ES*RS* ~ MAS* ~ GOV	1	0.230769
	~ NET* ~ OPL*FOD* ~ ES*RS* ~ MAS	1	0.230769
	NET*OPL*FOD*ES* ~ MAS* ~ GOV	1	0.230769
	NET*OPL* ~ FOD* ~ ES*RS*MAS	1	0.153846
	NET*OPL* ~ FOD* ~ ES*RS*GOV	1	0.153846
Minimalist path	~ FOD* ~ ES	1	0.307692
	~ NET* ~ ES	1	0.538462
	ES* ~ GOV	1	0.230769
	NET*ES	1	0.230769
	OPL*ES	1	0.230769
	FOD*ES	1	0.230769
	~ OPL* ~ ES* ~ MAS	1	0.538462

Data source: Translated and compiled by the author from the output of data analysis software

Among them, the complex path combination and the intermediate path combination remain consistent because no directional precisions are made when making counterfactual judgments, so the intermediate solution does not incorporate the logical residual term and the intermediate path obtained is consistent with the complex path. The conclusions obtained from the intermediate solutions between the complex and intermediate solutions are usually considered to be better revelatory and universal, and are optimal for reporting and interpretation in QCA studies. The consistency of the solutions of these four intermediate path combinations in this study is 1, which constitutes a sufficient necessary condition for multiple reversals of public opinion, implying that these path combinations can explain the cases of public opinion reversals in this study, and thus, the results of this analysis are strongly robust, where the original coverage of the first NET*OPL*FOD* ~ ES*RS* ~ MAS is 0.411765, indicating that the conditional path combination of strong relevance of Internet users and strong engagement of opinion leaders and graphic form and local scope and non-self-media and media without agenda setting can explain 41% of the cases. The original coverage of the second path NET*OPL*FOD* ~ ES*MAS*GOV is 0.470588, indicating that the combination of the conditional paths of strong netizen relevance and strong opinion leader involvement and graphic form transmission and local scope and media agenda setting and government department involvement can explain 47% of the cases. The third NET*FOD* ~ ES*RS*MAS*GOV has a raw coverage of 0.411765, which indicates that -this combination of conditional paths can explain 41% of the cases. The fourth path has a relatively low coverage of 0.0588235, which has limited explanatory power, so the first three intermediate paths are chosen for interpretation in this study. These three paths are parallel and equivalent to each other and can be expressed by the Boolean algebra “+” as

$$ \begin{aligned} {\text{RESULT}} & = {\text{NET}}*{\text{OPL}}*{\text{FOD}}*\sim {\text{ES}}*{\text{RS}}*\sim {\text{MAS}} + {\text{NET}}*{\text{OPL}}*{\text{FOD}}*\sim {\text{ES}}*{\text{MAS}}*{\text{GOV}} \\ & + {\text{NET}}*{\text{FOD}}*\sim {\text{ES}}*{\text{RS}}*{\text{MAS}}*{\text{ GOV}}{.} \\ \end{aligned} $$

After simplification, we get

$$ {\text{RESULT}} = {\text{NET}}*{\text{FOD}}*\sim {\text{ES}}*\left[ {{\text{MAS}}*{\text{GOV}}*\left( {{\text{OPL}} + {\text{RS}}} \right) + {\text{OPL}}**{\text{RS}}*\sim {\text{MAS}}} \right]. $$

To observe the results more intuitively and clearly, this paper identifies the core conditions in each driving path by comparing the nested relationship between the intermediate and parsimonious solutions of the QCA method, i.e., the condition variables that appear in both the intermediate and parsimonious paths are the core conditions of that path, and the conditions that appear only in the intermediate path are the peripheral conditions [20]. As in Table 8, “●” indicates that the condition is a “core condition and occurs”; “•” indicates that the condition is a “peripheral condition and occurs”; “⨂” indicates that the condition is "core condition and does not occur"; “^⨂^” indicates that the condition is "peripheral condition and does not occur"; blank indicates that "occurrence or non-occurrence has no effect on the result". Among them, there are three paths (S1, S2, S3) that drive the polycentric reversal of public opinion, and their core conditions are the same, i.e., strong relevance of netizens, dissemination in graphic form, and localized outbreak. Each combination of paths affecting the polycentric reversal of public opinion is analyzed in detail below.

**Table 8:** Combination of conditions for multiple and single center reversals of public opinion in fsQCA

Variables	Polycentric inverse path			Single center inversion path					
	S1	S2	S3	NS1	NS2	NS3	NS4	NS5	NS6
NET	●	●	●	⨂	⨂	⨂	●	•	•
OPL	•	•		⨂	⨂	⨂	●	•	•
FOD	●	●	●	•		•	●	⨂	⨂
ES	⨂	⨂	⨂	⨂	⨂	⨂	●	⨂	⨂
RS	•		•		•	•		•	•
MAS	^⨂^	•	•	⨂	⨂	⨂	^⨂^	•	
GOV		•	•	^⨂^	^⨂^		⨂		•
CS	1	1	1	1	1	1	1	1	1
CV	0.412	0.471	0.412	0.385	0.231	0.231	0.231	0.154	0.154

Note: CS denotes consistency and CV denotes coverage. Data source: Translated and compiled by the author from the output of data analysis software

#### Driving Path One: Opinion Leader–Media Dual-Driven Path

The first driving path is the opinion leader–media dual-driven path, and the path is represented as NET*OPL*FOD* ~ ES*RS* ~ MAS, which means that the possibility of polycentric reversal of public opinion is very high when these conditions are satisfied simultaneously: strong relevance of netizens, strong involvement of opinion leaders, graphic form, local scope of events and non-self-media channels, and no agenda setting of media.

#### Driving Path Two: Opinion Leader–Media–Government Multi-driven Path

The second driving path is the opinion leader–media–government multi-driven path, which is denoted as NET*OPL*FOD* ~ ES*MAS*GOV, implying that the possibility of polycentric reversal of public opinion is very high when these conditions are satisfied simultaneously: strong relevance of netizens, strong participation of opinion leaders, transmission of graphic form and local scope, and media agenda setting and government involvement.

#### Driving Path Three: Media–Government Dual-Driven Path

The third driving path is the media–government dual-driven path, denoted as NET*FOD* ~ ES*RS*MAS*GOV, where the possibility of polycentric reversal of public opinion is great when these conditions are satisfied simultaneously: strong relevance of netizens, graphic form, local scope, non-self-media, and media agenda setting and government involvement.

### Robustness Tests

Robustness testing of the analysis results is a key step in conducting QCA research, and there are various ways to test them. Generally, we can change the minimum case frequency and the consistency threshold into logical minimization to assess whether the analysis results are stable and reliable. In this paper, we adopt increasing the consistency level to test the robustness of public opinion polycentric reversal results. Specifically, the consistency threshold is adjusted from 0.8 to 0.9, and the number of grouping relationships and grouping paths does not change, indicating that the analysis results are robust.

## Conclusion and Discussion

### Conclusion

(1) Summary of factors influencing the reversal of online public opinion on sudden events.

The QCA method was used to test the univariate necessary conditions for these seven conditional variables and their respective inverse values, and the three necessary conditions leading to the results were the relevance of netizens, the form of public opinion dissemination, and the ~ scale of the event. In terms of netizen relevance, its consistency and coverage rate are 0.94118 and 0.72727, respectively, which indicates that the stronger the netizen relevance, the greater the possibility of reversal of public opinion. It is not difficult to understand that when the event involves personal safety, netizens will become concerned and sensitive, especially in the early stage of reversal of the event, most of the related reported information is dominated by sensitive information. Because it is closely related to their own safety and the safety of the society they live in, the thirst for the truth will drive the media and the government to reveal the truth about the incident, thus satisfying people's curiosity. In terms of the forms of public opinion dissemination, the consistency and coverage rates of graphic forms of dissemination are 0.94118 and 0.64000, respectively, which indicates that graphic forms of dissemination are more likely to drive public opinion to make multiple reversals. This is mainly related to the lower authenticity of the graphic form than the video form, which requires less faking cost and skills and is easier to produce and edit. This is mainly related to the misbehavior of communication under self-media. Currently, in the era of everyone has a microphone, everyone has the right to speak, and people are able to get traffic and attention, and publish relevant inaccurate information with graphics on self-media platforms such as Weibo and WeChat to get clicks before the mainstream media, which often leads to a small reversal of events at the early stage of the incident. In terms of the scale of events, the consistency and coverage rates of events occurring on a local scale are 0.94118 and 0.61539, respectively, which means that reversal events have a tendency to spread on a local scale. This is mainly related to the extent and impact of the event, which is directly related to the number of audiences; the smaller the number of audiences, the faster the information spreads, and every change and reversal of information is likely to cause one reversal of the event opinion.

(2) Summary of the driving paths of the reversal of online public opinion on sudden events.

This paper uses QCA analysis to explore the multiple concurrent cause–effect complex mechanism of unexpected online opinion reversal. By examining 30 unexpected reversal news events, three parallel and explanatory driving paths of multiple factor combinations leading to polycentric reversal of public opinion are derived: (1) Opinion leader–media dual-driven (NET*OPL*FOD* ~ ES*RS* ~ MAS): strong relevance of netizens and strong involvement of opinion leaders and graphic form and local scope events and non-self-media channels reversal and no agenda setting by media. (2) Opinion leader–media–government multi-driven path (NET*OPL*FOD* ~ ES*MAS*GOV): strong relevance of netizens and strong participation of opinion leaders, graphic form and local scope and media agenda setting and government department intervention; (3) media–government dual-driven path (NET*FOD* ~ ES*RS*MAS*GOV); strong relevance of netizens and graphic form and local scope and non-self-media and media agenda setting and government department intervention.

From the combination analysis of the three driving paths derived above, it is found that the seven conditional variables proposed in this paper based on the factors affecting public opinion reversal (relevance of netizens, involvement of opinion leaders, form of public opinion dissemination, scale of events, reversal channels, media agenda setting, and government department involvement) all appear in different driving paths, which can indicate that all seven conditional variables have some degree of influence on the generation of outcome variables. Among these three driving paths, especially the original coverage rate of the second opinion leader-media-government driven path is 47%, which is close to 50%, and the original coverage rates of the first opinion leader–media driven path and the second media-government driven path are both higher than 40%, indicating that the combination of these three paths can explain most of the opinion reversal cases, and thus has strong explanatory power and applicability. After simplifying the three driving paths, we can get: NET*FOD* ~ ES*[MAS*GOV*(OPL + RS) + OPL*RS* ~ MAS], and after translation, we can find that: strong relevance of netizens and dissemination in graphic form with local scope and (media agenda setting and government department intervention and (high involvement of opinion leaders or non-self-media reversal) or high involvement of opinion leaders and non-self (media reversal and no agenda setting by media), that is, the combination of any one of the two conditions with the previous three conditions will lead to the outbreak of public opinion reversal, so it seems that the combination of the three triggering factors, namely strong relevance of netizens and dissemination in graphic form and localized occurrence, is very important to lead to public opinion reversal.

### Discussion

(1) The public: improve media literacy and journalistic taste, and maintain a return to rationality.

The information audience side, as the disseminator and receiver of online public opinion information, and netizens, as a huge group in online public opinion, are the main participants in the reversal of public opinion. It is crucial to improve Internet users' own media literacy. Internet users are required to have certain judgment ability and should keep a clear head in the face of the news information reported by the media. Even in the face of official media and authoritative media information dissemination, there may be information distortion. Netizens should maintain a reasonable skeptical attitude and not blindly follow the trend. In the reversal of public opinion on the Internet, the audience should try to reject blind communication, not to take media reports as a guideline, but to dare to question the media, to keep calm and think about the content reported by the media.

(2) Media: Raising the awareness and consciousness of "gatekeepers" and building an objective issue framework.

When the media deliberately create and publish relevant reports to meet people's stereotypes and labeling psychology, they not only spread misinformation but also carry out misguidance, seriously ignoring the professional ethics code that the media itself should have; mainstream media should adhere to journalistic professionalism and break the multiple information fogs. Communication and interaction between mainstream media and Internet users are indispensable, especially when it comes to the reversal of public opinion, the relationship between the two should be presented as a benign interaction process. In this process, mainstream media should have a sense of social responsibility, give full play to their advantage of "gatekeeper", filter and control the truth and falsity of news content, so as to set the tone in the development of public opinion.

(3) Government: establish a sense of active communication, regulation and guidance in parallel.

In response to public opinion events, government platforms should establish a sense of initiative, proactively cooperate with the media and investigate the truth of the events. We need to provide timely and effective explanations for false news and rumors in cyberspace, and answer questions from the public at the first opportunity. Social media has dramatically changed the way the public communicates with government departments, and government microblogs have become the preferred communication channel. Mobile social media, such as microblogs, are efficient and accessible for information dissemination, which can improve the distance between the government and the public, thus promoting a positive interaction between them. An important condition to avoid frequent reversal of public opinion is that the government should have enough credibility, communication power and influence when releasing information. Government platforms should actively and timely release some high-quality exclusive information with the help of social microblogs such as government microblogs, and make the information as transparent as possible.

## Data Availability

The datasets used and/or analyzed during the current study are available from the corresponding author on reasonable request.

## Abbreviation

QCA: Qualitative comparative analysis
